# How Live Music Moves Us: Head Movement Differences in Audiences to Live Versus Recorded Music

**DOI:** 10.3389/fpsyg.2018.02682

**Published:** 2019-01-11

**Authors:** Dana Swarbrick, Dan Bosnyak, Steven R. Livingstone, Jotthi Bansal, Susan Marsh-Rollo, Matthew H. Woolhouse, Laurel J. Trainor

**Affiliations:** ^1^Department of Psychology, Neuroscience & Behaviour, McMaster University, Hamilton, ON, Canada; ^2^McMaster Institute for Music and the Mind, McMaster University, Hamilton, ON, Canada; ^3^Digital Music Lab, School of the Arts, McMaster University, Hamilton, ON, Canada; ^4^Rotman Research Institute, Baycrest Hospital, Toronto, ON, Canada

**Keywords:** live concert, recorded music, music, fan, entrainment, movement, motion capture

## Abstract

A live music concert is a pleasurable social event that is among the most visceral and memorable forms of musical engagement. But what inspires listeners to attend concerts, sometimes at great expense, when they could listen to recordings at home? An iconic aspect of popular concerts is engaging with other audience members through moving to the music. Head movements, in particular, reflect emotion and have social consequences when experienced with others. Previous studies have explored the affiliative social engagement experienced among people moving together to music. But live concerts have other features that might also be important, such as that during a live performance the music unfolds in a unique and not predetermined way, potentially increasing anticipation and feelings of involvement for the audience. Being in the same space as the musicians might also be exciting. Here we controlled for simply being in an audience to examine whether factors inherent to live performance contribute to the concert experience. We used motion capture to compare head movement responses at a live album release concert featuring Canadian rock star Ian Fletcher Thornley, and at a concert without the performers where the same songs were played from the recorded album. We also examined effects of a prior connection with the performers by comparing fans and neutral-listeners, while controlling for familiarity with the songs, as the album had not yet been released. Head movements were faster during the live concert than the album-playback concert. Self-reported fans moved faster and exhibited greater levels of rhythmic entrainment than neutral-listeners. These results indicate that live music engages listeners to a greater extent than pre-recorded music and that a pre-existing admiration for the performers also leads to higher engagement.

## Introduction

Music is a universal social phenomenon that has traditionally been experienced in a live context (Nettl and Russell, 1998; Freeman, 2000). The advent of recording technology in the late 19th century heralded a cultural shift in the way that people experienced music, allowing for the convenience of private, in-home consumption (Moreau, 2013). While technology has provided a low-cost, convenient method for music listening, many people continue to attend live concerts, sometimes at great expense in uncomfortable settings (Baxter-Moore and Kitts, 2016; Brown and Knox, 2017). What is it about the experience that motivates listeners to attend live concerts? A survey found that listeners’ strongest musical experiences often took place at live events (Lamont, 2011). Two factors that likely contribute critically to the enjoyment of live concerts are (1) people like the social connexion of experiencing music with other people (Burland and Pitts, 2014; Brown and Knox, 2017) and (2) people like the feeling of being connected to the performers, by being in the same physical space together, with the potential for performers to directly engage the audience (Silverberg et al., 2013; Leante, 2016), and by experiencing a unique live performance as it unfolds over time (Brown and Knox, 2017). Every live performance is idiosyncratic such that events unfold organically and unpredictably, unlike when listening to a recording in which there is no possibility for an audience to directly affect what a performer has already created.

The social effects of experiencing music with other people have been studied to a greater extent than the effects of experiencing a live performance (Freeman, 2000; Egermann et al., 2011; Rennung and Goritz, 2016; Stupacher et al., 2017). Here we examined the effects of live performance while controlling for the social setting. We compared people who listened to a live performance (specifically, a record release party by Canadian rock star Ian Fletcher Thornley’s 2015 solo album *Secrets*) to people who listened in a group in the same venue without live performers to the album recordings of the same songs from *Secrets*. Recently, research on audiences of live performances has gained interest (Egermann et al., 2011; Burland and Pitts, 2014; Danielsen and Helseth, 2016; Bradby, 2017; Brown and Knox, 2017), in part because audiences provide an ecologically valid setting for examining group dynamics. Audience experience has been examined with a variety of techniques including real-time subjective responses (McAdams, 2004; Stevens et al., 2009, 2014; Egermann et al., 2013), social networking (Deller, 2011), video analysis (Chan et al., 2013; Silverberg et al., 2013; Stevens et al., 2014; Leante, 2016; Theodorou et al., 2016) and physiological measurement (Fancourt and Williamon, 2016; Bernardi et al., 2017). It is important to understand effects of the concert setting because attendance may increase health: attending a musical performance was found to reduce stress hormones in audience members (Fancourt and Williamon, 2016) and a 10-year longitudinal study suggested that engagement in cultural events, including concerts, may protect against age-related cognitive decline (Fancourt and Steptoe, 2018).

Enjoying music with other listeners may contribute powerfully to the concert experience. Observers of concert audiences judged synchronously moving listeners as experiencing greater rapport and similar psychological states compared to those moving asynchronously (Lakens and Stel, 2011). After adults move in synchrony, even when unaware of their synchronised movements, they remember more about each other, express liking each other more, and show greater levels of trust and cooperation compared to after moving asynchronously (Hove and Risen, 2009; Wiltermuth and Heath, 2009; Valdesolo et al., 2010; Valdesolo and DeSteno, 2011; Launay et al., 2013; Woolhouse et al., 2016). More broadly, periodic movements and physiological rhythms, such as breathing and heart rate, tend to synchronise unconsciously among people in a group (Richardson et al., 2007; van Ulzen et al., 2008; Morris, 2010; Codrons et al., 2014; Miyata et al., 2018).

Entrainment is defined as the ability to synchronise movements with an external auditory stimulus, in this case the timing regularities of music (Phillips-Silver and Keller, 2012). In humans, synchronisation is supported by connections between auditory and motor cortices (Sakai et al., 1999; Janata and Grafton, 2003; Grahn and Brett, 2007; Zatorre et al., 2007; Fujioka et al., 2012) and manifests as oscillatory activity measured in EEG and MEG (Schroeder and Lakatos, 2009; Arnal and Giraud, 2012; Fujioka et al., 2012, 2015; Cravo et al., 2013; Calderone et al., 2014; Cirelli et al., 2014a; Chang et al., 2018a). Interestingly, few non-human species entrain movements to auditory regularities (Merker et al., 2009; Patel et al., 2009; Schachner et al., 2009). The connection between movement synchronisation and social-emotional engagement may have deep evolutionary roots in humans. Infants are not yet able to coordinate their movements to entrain to a musical beat, although they do move faster to music with a faster compared to slower tempo (Zentner and Eerola, 2010). Yet if an infant as young as 14 months is bounced to music synchronously with the movements of another adult, the infant is more likely to help that adult (e.g., to pick up “accidentally” dropped objects needed to complete a task) compared to if an infant is bounced asynchronously with the adult (Cirelli et al., 2014c). Later work revealed that this increased helpfulness extends to friends of the experimenter who bounced with them (Cirelli et al., 2016). In another study, infants who were bounced to music with stuffed animals, choose animals that bounced synchronously with them over animals that bounced asynchronously. These studies indicate that synchronisation of movement with others during music listening is a cue that even infants use in the development of social-emotional bonds and altruistic behaviours (Trainor and Cirelli, 2015; Cirelli et al., 2018).

We examined the effect of live music while controlling for the effects of being with others in an audience. Little research has examined differences between live and recorded performances by manipulating the presence and absence of the performer. Shoda et al. (2016) reported that the heartbeats of audience members at a live performance exhibited greater entrainment with the musical rhythm than those of listeners at a pre-recorded performance. Performer presence was also found to produce greater relaxation in audience members compared to those listening to a recording (Shoda et al., 2016). Contemporary popular performers often play variations of recorded works at live performances (Shoda and Adachi, 2015), suggesting a novelty factor for listeners. Brown and Knox (2017) found that audience members consider this musical novelty as an important motivator for concert attendance. Live concerts also enable audience members to experience an in-person relationship with the performer. Performers can also be influenced by the presence of an audience, and live performances can be acoustically and energetically different than those recorded in the studio (Zajonc, 1965; Yoshie et al., 2016; Bradby, 2017).

We used head movement responses as our main measure of audience experience for several reasons. Moving to the beat during music listening is culturally ubiquitous, with collective movement a hallmark of the contemporary concert experience (Zatorre et al., 2007; Madison et al., 2011; Janata et al., 2012; Davies et al., 2013; Madison and Sioros, 2014; Stupacher et al., 2017). Individuals use a range of movements when listening to music, from foot tapping to head nodding, to whole body movement (Leman and Godøy, 2010). Head movements are particularly relevant as they are a reliable indicator of rhythmic entrainment (Toiviainen et al., 2010; Burger et al., 2013), reveal communication patterns between performers (Chang et al., 2017), reveal directional and emotional communication patterns (Chang et al., unpublished), and even predict who will “match” during speed dating (Chang et al., 2018b). Movement of the head alone—but not legs alone—affects how ambiguous auditorily-presented rhythms are interpreted (Phillips-Silver and Trainor, 2008). This interaction between head movement and auditory perception likely involves the vestibular system located in the inner ear which processes proprioceptive information about head movements (Trainor et al., 2009). Head movements also encode emotional information (Livingstone and Palmer, 2016; Chang et al., unpublished), and may function as a form of non-verbal communication in a noisy environment (Harrigan et al., 2008). Head movements provide information about the nature of an emotion being communicated (Ekman and Friesen, 1967; Witkower and Tracy, 2018). Furthermore, movement smoothness (which increases with movement speed) is greater when communicating joy than a neutral emotion or sadness (Kang and Gross, 2016). Horizontal head movements and forward velocity communicate happiness even without the context provided by facial expression or vocal content (Livingstone and Palmer, 2016). Additionally, movement vigour (average speed) and movement distance have been shown to convey the intensity of emotions (Atkinson et al., 2004). Leow et al. (2015) found that, even when asked to walk at the same tempo, participants walked more vigorously (faster) to more familiar music. One study found that during music listening, greater head speed was correlated with increased spectral flux in low frequencies (associated with greater presence of kick drum and bass guitar) and in high frequencies (associated with hi-hat and cymbals or liveliness of a rhythm), as well as with greater percussiveness, but head speed was not found to be related to tempo (Burger et al., 2013).

In summary, there are many possible factors contributing to movement during music listening including biological imperatives, emotions, and the presence of others. These factors have been studied in highly controlled laboratory settings but have yet to be explored in real-world music listening contexts. In the present study, we were interested in how a live concert affected audience head movements as an index of engagement, specifically, by comparing the movements of concertgoers who experienced a live performance versus a recorded version of the same songs. We were particularly interested in the measure of vigour. Following previous researchers, we operationally defined movement vigour as the average speed of movement over a time interval, regardless of direction (specifically, head distance travelled within a song divided by the total length of the song, giving a value in millimetres per second) (Atkinson et al., 2004; Mazzoni et al., 2007; Zentner and Eerola, 2010). We were also interested in how head movements might be influenced by audience members’ prior admiration for the performers (i.e., their Listener-preference). People are motivated to attend music concerts when they hold a strong preference for the musicians’ work. Musical preferences for genres and artists also play a role in defining social affiliations, particularly during adolescence, where they appear to function as a ‘badge of identity’ within a social group (North and Hargreaves, 1999; Mulder et al., 2010). ‘Fans’ of a particular performer would be expected to enjoy musical performances by that performer, in part because the familiarity gained from repeated exposure to recordings of their music would be expected to increase enjoyment of the performer’s music in general (Schellenberg et al., 2008; van den Bosch et al., 2013). To examine the effect of audience members’ prior preferences for the band, we recruited fans of the performer Ian Fletcher Thornley, along with naïve listeners who expressed no particular preference for the performer. Since the album had not yet been released prior to the concerts, the effects of song familiarity were controlled while examining differences between fans and neutral listeners as neither group had heard the songs prior to the concerts.

In sum, we examined the effects of live versus pre-recorded music and fan status on audience engagement with the music through head movements. Self-reported Fans and Neutral-preference listeners were separately recruited, and randomly assigned to attend one of two concerts. The concerts served as the record release event for Canadian rock star Ian Fletcher Thornley’s 2015 solo album *Secrets*, featuring new unreleased music. In the Live concert, audience members experienced a live performance by the musicians, while in the Album-playback concert, listeners heard an audio recording of the same songs from the *Secrets* album. Both concerts were held in the LIVELab, a 106-seat performance hall equipped with a 25-camera optical motion capture system. Head movements of participants were recorded simultaneously throughout each of the two concerts (Supplementary Figure S1). Two aspects of head movement were examined: (1) vigour and (2) entrainment to the beat of the music. We hypothesised that head movements would be faster and better entrained when audiences experienced a live concert compared to a pre-recorded version of the music. We further hypothesised that fans of the performer would exhibit faster movement, and entrain better to the rhythm, compared to neutral listeners.

## Materials and Methods

### Participants

Fans of the performer were recruited through contests advertised in social media (*n* = 39). Neutral-listeners who expressed no specific preference for Ian Fletcher Thornley (*n* = 21) were recruited for course credit through McMaster University’s online research portal (*n* = 3), social media and flyers circulated across campus and in music stores (*n* = 18). Self-asserted Fan-status was verified via a follow-up questionnaire. Participants’ demographics and condition assignments are described in Table 1. Prior to analysis, five participants were excluded due to: self-reported abnormal hearing (*n* = 1 from Live/Neutral-listener condition), movement restrictions (*n* = 1 from Album-playback/Fan condition), or having previously heard songs from the album (*n* = 3; 1 from Album-playback/Fan, 2 from Live/Fan conditions). Six participants who did not respond to a follow-up survey confirming fan-status were further excluded: 1 from Album playback/Fan, 2 from Album-playback/Neutral-listener, 2 from Live/Fan, 1 from Live/Neutral-listener conditions. The final sample consisted of 32 Fans and 17 Neutral-listeners. The McMaster University Research Ethics Board approved all procedures.

**Table 1:** Participant demographics.

Performer condition	Listener preference	*N*	Gender (female, male)	Mean age (years)	Age range
Present (live concert)	Fans	15	7, 8	39.7	28–53
	Neutral	9	3, 6	25.2	19–50
Absent (pre-recorded)	Fans	17	7, 10	31.8	19–51
	Neutral	8	5, 3	29.8	19–57
Total		49	22, 27	31.63	19–57

### Stimuli and Apparatus

Ian Fletcher Thornley’s record release party concert was the setting for this study. Participants listened to eight songs from Thornley’s new studio album *Secrets* on the day of its official release. This release reached a top position of 9 on the Canadian iTunes sales charts on October 30th, 2015. The first seven songs were novel to all included participants. The final song in the concert, “Blown Wide Open,” was a cover version of a previous song that was familiar to fans^1^. The eight songs were presented in the following order in both conditions: (1) “Just to Know I Can”; (2) “How Long”; (3) “Fool”; (4) “Elouise”; (5) “Frozen Pond”; (6) “Feel”; (7) “Secrets”; and (8) reinterpretation of “Blown Wide Open”. These stimuli are hereafter referred to as Songs 1 through 8, respectively.

Both the Live and Album-playback concerts took place in the LIVELab^2^. The LIVELab is a research facility with a 106-seat performance hall designed for the study of human interaction in a variety of ecologically valid contexts, including music, dance and pedagogy. In both Live and Album-playback concerts, motion-recorded Fans and Neutral-listeners were seated interspersed in the front and centre of the audience across four rows with an average of 8 people per row. Sound for both concerts was presented over a high-quality Meyer Sound 6 channel house PA system (Left/Right Main Speakers, Meyer UPJ, Left/Right Front Fill, Meyer UP4, Left/Right Subwoofer, Meyer 500-HP). Reverberation was added to each instrument in the Live Concert via a Digico SD9 sound mixer. A sound technician manipulated volume and reverberation throughout the live concert as it would be at a professional live show. For the Live Concert condition, Thornley (vocals and electric guitar) and his band (electric bass, drums, and cigar box guitar) performed renditions of the 8 songs in the same order as they were presented in the Album-playback concert condition. Given that it was a live performance, there were minor variations in tempo and arrangement between the stimuli at the Live compared to Album-playback Concerts, as would be expected in any live performance of a recorded work (see Supplementary Table S1 in the Supplementary Material for a comparison of the tempi of the pre-recorded and live songs). Coloured stage lights helped create the concert experience. Videos depicting a variety of neuroscience-themed phenomena played behind the performers on the stage video wall (3 × 3 array of Mitsubishi LM55S 55″ monitors) during the Live concert. In song 6, “Feel,” a video depiction of a previous recording of Thornley’s neural responses when listening to the recording of his own song “Feel” were imaged from fMRI and EEG data. Referred to as “Lightning Brain,” the 5-min video can be viewed online^3^.

In the Album-playback concert, a photo of the *Secrets* album artwork was displayed on the stage video wall and the stage was dimly lit with coloured lights. The stage setup was identical for the two conditions; all of the instruments were in place and ready for performance. During Song 6 the video depiction of Thornley’s neural responses was displayed as in the Live concert. See Supplementary Table S1 for the tempi of the recorded and live songs.

### Design and Procedure

The experimental design was a 2 × 2 × 7 with between-subjects factors Concert-status (Live, Album-playback) and Listener-preference (Fan, Neutral-listener) and within-subject factor Song (1, 2, 3, 4, 5, 6, and 7). The 8th song was analysed separately with only the between-subjects factors since it was familiar to Fans.

Fans and Neutral-listeners were randomly assigned to the Live or Album-playback conditions. In both cases participants were greeted at the entrance, filled out a consent form, and were fitted with a motion-capture cap. The caps did not restrict listener movement in any way. Participants were ushered into the theatre and to their seat. Once seated, additional audience members who did not participate in the study were then admitted to the theatre. Two researchers thanked the participants for their attendance and introduced the concert. Participants were instructed to do their best to forget that they were wearing caps and to enjoy the concert as they normally would. They were given no further instructions and were not encouraged to move in any particular way. Participants then completed a questionnaire on their familiarity with the performers, their current state of arousal and happiness, and their musical expertise (see Appendix S1 in Supplementary Material). A follow-up questionnaire at the end of the concert asked the same questions regarding listener arousal and happiness.

Both concerts (Live, Album-playback) took place on the same day, with the Album-playback concert in the afternoon and the Live concert in the evening. During the Live concert, Thornley occasionally spoke to the audience between songs as performers would at a typical concert. Head movements between songs were not analysed. At the end of the Album-playback condition, Thornley and his band played a live song to avoid disappointing fans; head motion during this song was not analysed. A second questionnaire was sent to participants after the experiment to collect participant demographic information including age, sex, detailed music and dance experience and preferences.

### Data Recording and Analysis

An audio recording of the live performance was recorded for later analysis. A passive optical motion capture system (24 Oqus 5+ cameras and an Oqus 210c video camera, Qualisys) recorded the head movements of participants at 90 Hz. Four retroreflective markers (10 mm) were placed on felt caps worn by the participants, forming a rigid body. One marker was placed on the front of the head, one on top of the head, and one on each temple.

Motion capture data were cleaned and labelled using the Qualisys Track Manager, then exported to MATLAB (The MathWorks Inc., 2015) for analysis with the motion capture toolbox (Burger and Toiviainen, 2013). Motion data were gap-filled using linear interpolation, then low-pass filtered at 6 Hz to remove jitter. The positions of the four head markers were averaged to produce a single, stable representation of participant head centre (Supplementary Figure S1). Data were then normalised and segmented into songs. After preparation, two measures of participant head motion were generated.

#### Movement Vigour

The average movement speed of each participant in mm/s was calculated to provide a representation of movement vigour (Mazzoni et al., 2007; Zentner and Eerola, 2010; Leow et al., 2014). The speed of participants’ movements was estimated by taking the first derivative of the motion signal (differences in position between adjacent frames). Speed trajectories were then smoothed using a second-order lowpass Butterworth filtre with a normalised low-pass frequency of 0.2π radians per sample. At a sampling frequency of 90 Hz, this equated to a 9 Hz low-pass filtre. Movement vigour is conceptually independent of synchronisation; a participant could remain in perfect synchrony to a given tempo and still move with more or less vigour (e.g., by increasing or decreasing the distance they moved their head), and a participant could also remain completely unsynchronised and still move with more or less vigour.

#### Degree of Entrainment

The degree of entrainment was defined as how frequently participants entrained their movements to the beat of each song. Movement periodicities were extracted with a windowed autocorrelation performed on listeners head-centre motion trajectories, with window size of 10 s, hop size of 5 s, and lags ranging from 0 to 2 s using *mcwindow* and *mcperiod* functions from the Mocap Toolbox (Eerola et al., 2006; Burger and Toiviainen, 2013). The tempi of the songs from both the Live and Album-playback concerts were determined by two musically trained raters (first and third authors, *n* = 9 and *n* = 15 years of formal training, respectively) who tapped along to the beat of each song while listening to the recordings of the album and the Live concert using a metronome application (Metronome Beats, Stonekick©2015). The average inter-beat interval period was calculated from the song tempo, and this period was used to calculate the period at the quarter, half, and whole note levels of the musical metrical hierarchy for each song at which participants could have entrained. The participants’ head movement period at each window, obtained from the autocorrelation analysis, was compared to the three possible periods of each song. If the participant’s period of motion was within 5% of one of these beat periods, then that window was added to a count of the number of windows demonstrating entrainment. The measure of degree of entrainment was defined as the number of windows with entrainment divided by the total number of possible windows, to give the proportion of entrainment, which could range between 0.0 (no entrainment) and 1.0 (perfect entrainment). Actual measured proportions ranged from 0.0 to 0.58 depending on the participant and song. Our overall grand mean entrainment proportion of 0.081 was smaller, but of similar magnitude, to that found by Burger et al. (2014) who showed period-locking proportions less than 0.3 (summing tactus divisions and excluding inferior-superior movement, which our seated participants were not free to engage in). Smaller values would be expected in our case, given that for the Burger et al. (2014) experiment participants were standing and specifically asked to move to the music, whereas in the present study participants were seated and were not given any instructions regarding movement.

## Results

### Analyses of the First Seven Unfamiliar Songs

Movement vigour and degree of entrainment were analysed with repeated measures ANOVAs, with between-subjects factors Concert-status (Live, Album-playback) and Listener-preference (Fan, Neutral-listener), and within-subjects factor Song (1, 2, 3, 4, 5, 6, and 7). When Mauchly’s test indicated that sphericity was violated, Greenhouse-Geisser’s corrections were applied. Effect sizes are reported with partial eta-squared values, means are accompanied by a variance measure of one standard error of the mean (*SEM*). Pairwise comparisons were adjusted using Bonferroni correction. Statistical tests were conducted in SPSS 2013 v20.0.0. Experiment-wise corrections were not implemented on the reported values, but below we note the two cases in which such a correction would affect interpretation of an effect as significant.

#### Concert-Status

There was a main effect of Concert-status for vigour, but not for entrainment, *F*(1,45) = 15.783, *p* < 0.001, $\eta_{p}^{2}$ = 0.260 and *F*(1,45) = 1.569, *p* = 0.217, $\eta_{p}^{2}$ = 0.034, respectively. Participants moved more vigorously in the Live concert (*M* = 15.559, *SEM* = 1.397) than the Album-playback concert (*M* = 7.644, *SEM* = 1.421) condition. These results indicate that the Live concert increased vigour but not necessarily the degree of entrainment of head movements. The interaction between Concert-status and Listener-preference was not significant for either vigour or entrainment.

#### Listener-Preference

As predicted, there was a main effect of Listener-preference for both vigour and entrainment, *F*(1,45) = 12.871, *p* = 0.001, $\eta_{p}^{2}$ = 0.222, and *F*(1,45) = 4.197, *p* = 0.046, $\eta_{p}^{2}$ = 0.085, respectively. (Note that the effect of Listener-preference on entrainment is no longer significant if experiment-wise Bonferroni correction for multiple comparisons is implemented). Fans (*M* = 15.175, *SEM* = 1.174) moved faster than Neutral-listeners (*M* = 8.027, *SEM* = 1.610) and Fans (*M* = 0.074, *SEM* = 0.007) showed a higher degree of entrainment than Neutral-listeners (*M* = 0.050, *SEM* = 0.01). These results indicate that Listener-preference affected both vigour and entrainment of head movements. The interaction between Concert-status and Listener-preference was not significant for either vigour or entrainment.

#### Song

**FIGURE 1:**
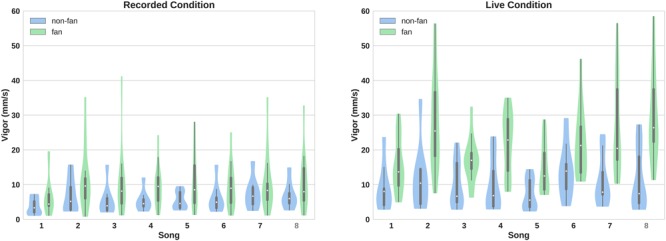
Vigour of head movements across songs. The distance travelled within a song was divided by the total length of the song, giving a value in millimetres per second. Fans moved with greater vigour than Neutral-listeners for every song and those in the Live Concert condition moved with greater vigour than those in the Album-playback Concert condition for every song. Vigour varied among songs, and was qualified depending on Concert-status (Live, Album-playback). The songs were: (1) “Just to Know I Can”; (2) “How Long”; (3) “Fool”; (4) “Elouise”; (5) “Frozen Pond”; (6) “Feel”; (7) “Secrets”; and (8) reinterpretation of “Blown Wide Open.” The violin plots show the same parameters as a standard box plot (range, interquartile range and median) as well as a kernel density plot that estimates the continuous distribution of the data.

**FIGURE 2:**
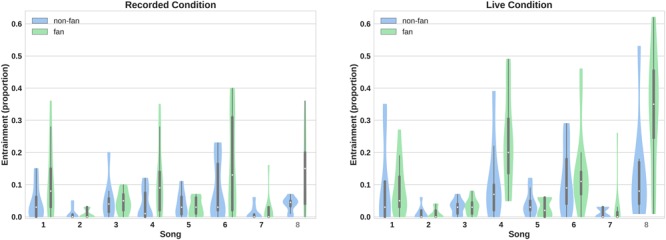
Proportion of movement entrainment across songs. Fans generally showed a higher degree of entrainment to the tempo of the music than Neutral-listeners. However, there was variation among songs, which interacted with Concert-status. The songs were: (1) “Just to Know I Can”; (2) “How Long”; (3) “Fool”; (4) “Elouise”; (5) “Frozen Pond”; (6) “Feel”; (7) “Secrets”; and (8) reinterpretation of “Blown Wide Open.” The violin plots show the same parameters as a standard box plot (range, interquartile range and median) as well as a kernel density plot that estimates the continuous distribution of the data.

In addition to the main effects produced by the between-subjects variables, there was a main effect of Song for both vigour and entrainment, *F*(4.439,199.768) = 9.626, *p* < 0.001, $\eta_{p}^{2}$ = 0.176 and *F*(3.254,146.414) = 19.022, *p* < 0.001, $\eta_{p}^{2}$ = 0.297, respectively. This indicates substantial differences between songs in their ability to produce both fast and entrained movement, likely due to intrinsic properties of the songs, such as tempo (see Figures 1, 2; song tempi are provided in Supplementary Table S1 in the Supplementary Material). Interestingly, songs producing the fastest movement were not necessarily the same songs that produced maximal entrainment, indicating the possibility of some level of independence between these two measures. An acoustic analysis of the songs from both performances is underway as a separate paper in which we plan to relate head movements to characteristics such as Danceability, Energy, Instrumentalness, Liveness, and Valence of individual songs.

There was also an interaction between song and Listener-preference for both vigour and entrainment, *F*(4.439,199.768) = 2.428, *p* = 0.003, $\eta_{p}^{2}$ = 0.082, and *F*(3.254,146.414) = 3.010, *p* = 0.029, $\eta_{p}^{2}$ = 0.063, respectively. This interaction indicates that Fans and Neutral-listeners reacted differently to different songs (It should be noted that the interaction between song and Listener-preference on entrainment is no longer significant if experiment-wise Bonferroni correction for multiple comparisons is implemented).

### Analyses of the 8th Song

The final song (“Blown Wide Open,” released in 1997) was analysed separately because it was familiar to Thornley’s fans, having been one of the most famous songs from his previous band Big Wreck. This provides a preliminary exploration of how familiarity can promote movement.

#### Concert-Status

There was a main effect of Concert-status on vigour, *F*(1,45) = 16.929, *p* < 0.001, $\eta_{p}^{2}$ = 0.273. Movement was more vigorous in the Live concert (*M* = 20.32 mm/s, *SEM* = 2.003) than Album-playback concert (*M* = 8.56 mm/s, *SEM* = 2.037) condition. There was also a main effect on entrainment, *F*(1,45) = 11.917, *p* = 0.001, $\eta_{p}^{2}$ = 0.209. The degree of entrainment was higher in the Live concert (*M* = 0.235, SEM = 0.029) than Album-playback concert (*M* = 0.091, SEM = 0.030) condition.

#### Listener-Preference

For Listener-preference, there was a main effect on vigour, *F*(1,45) = 14.494, *p* < 0.001, $\eta_{p}^{2}$ = 0.244. Fans (*M* = 19.88 mm/s, *SEM* = 1.683) moved faster than Neutral-listeners (*M* = 9.00 mm/s, *SEM* = 2.308). There was also a main effect on entrainment, *F*(1,45) = 13.630, *p* = 0.001, $\eta_{p}^{2}$ = 0.232. Fans (*M* = 0.24, *SEM* = 0.025) entrained to a greater degree than Neutral-listeners (*M* = 0.086, *SEM* = 0.034). The interaction between Concert-status and Listener-preference was not significant.

### Musicians Versus Non-musicians

Using the self-reported measures of music experience, participants were categorised as musicians (*N* = 25; mean years of training = 11.7; range = 1–38) or non-musicians with no musical training (*N* = 24). Independent-samples *t*-tests were performed for vigour for the mean of Songs 1–7, *t*(47) = 0.6, *p* = 0.58, vigour for Song 8, *t*(47) = 0.4, *p* = 0.68, entrainment for the mean of Songs 1–7, *t*(47) = 0.5, *p* = 0.62, and entrainment for Song 8, *t*(47) = 0.8, *p* = 0.45. There were no significant differences on any of these measures.

## Discussion

The question of why people enjoy attending live concerts, when the same music can be experienced more easily and for less money at home, likely involves two aspects: the social sharing of the experience in a group of people; and “live” aspects, including connecting with the artists and experiencing the potential for spontaneity and unpredictability of live music as it unfolds over time, compared to a pre-recorded and unchanging version on a recording that a fan might become familiar with after repeated listening. In our study, we examined primarily the second aspect, comparing listening to a recording of a set of songs from Ian Fletcher Thornley’s 2015 album *Secrets* to listening to a live performance of those songs, while keeping the social aspect largely the same: both the Live and Album-playback concerts were experienced in the context of an audience in the same LIVELab venue. In the case of this study, audiences were not familiar with recorded songs, but nonetheless may have reacted to the knowledge that the music in the Live condition was unfolding in a unique way that would never be repeated exactly. Necessarily, the visual stimulation differed between the two conditions because of the presence of the live performers. We feel that this is not necessarily a confound—a live performance requires the presence of performers—but future studies might incorporate some visual stimulation that tries to better equate the two conditions, for example, by showing a video of the live performance. We also examined how being a fan of the musical group affected these experiences by comparing self-reported Fans and Neutral-listeners randomly assigned to the Live and Album-playback concert conditions. We focused on head movements, using motion capture to extract the vigour and degree of entrainment of head movements to the beat of music (Toiviainen et al., 2010; Burger et al., 2013).

We found that for both Fans and Neutral-listeners, head movements were more vigorous in the Live than the Album-playback concert, but Concert-status did not affect degree of entrainment to the beat. On the other hand, across both concert conditions, Fans moved their heads more vigorously and with better entrainment to the beat compared to Neutral-listeners. The greater degree of entrainment to the beat in general in Fans likely reflects their greater familiarity with the artist’s musical style. The greater vigour of head movements across groups at the Live compared to Album-playback concert likely represents greater arousal, increased anticipation, and increased connection with the artists and their music during the live concert (Mazzoni et al., 2007; Leow et al., 2014). Amount of musical training varied across audience members, but there were no differences between musicians and non-musicians in either movement vigour or synchronisation to the beat. Similarly, Bernardi et al. (2017) reported that musical training did not affect the degree of synchronisation of autonomic responses to the beat of music experienced in a group setting. Together, these results suggest that entrainment responses in audiences are independent of musical training.

We controlled for song familiarity across Fans and Neutral-listeners by using songs that had not yet been publicly released (the first 7 songs of the concerts). The eighth song, “Blown Wide Open,” on the other hand, was certainly familiar to Fans, and may have been familiar to some Neutral-listeners as its original rendition had achieved double platinum sales in Canada in the late 1990s. Interestingly, when the songs were not familiar, there was no difference in degree of entrainment to the music across the Live and Album-playback concerts. However, for the eighth song that was familiar at least to Fans, head movement entrainment was greater during the Live than Album-playback concert. This suggests that while the vigour of head movements is affected by whether the music is live or pre-recorded regardless of familiarity, familiarity with the music may foster greater entrainment to the beat during live compared to recorded contexts.

Vigour of head movements and degree of entrainment differed across songs. Further, there were interactions for both measures between Songs and Listener-preference, indicating that Fans and Neutral-listeners reacted differently to different songs. This suggests that some songs might excite existing fans differently than naïve listeners, which might inform record company promotion decisions. Concerts are becoming increasingly important for the music industry as the prevalence of piracy results in reduced revenue from album recordings (Frith, 2007; Papies and van Heerde, 2017). Interestingly, the majority of audience members report that cost does not influence their decisions to attend concerts (Brown and Knox, 2017). In general, research on audience development and retention could be important for sustaining the multi-billion dollar music industry (O’Reilly et al., 2014; Papies and van Heerde, 2017).

Music compels us to move, the likely result of connections between auditory and motor areas of the brain (Sakai et al., 1999; Janata and Grafton, 2003; Grahn and Brett, 2007; Zatorre et al., 2007; Grahn and Rowe, 2009; Janata et al., 2012), whose communication during rhythm and beat prediction can be measured in neural oscillations (Fujioka et al., 2012). Certain characteristics of music lead to increased entrainment to music and compulsion of movement, such as beat predictability and rhythmic complexity (Fitch, 2016), the density of events between beats (Madison et al., 2011), moderate levels of syncopation (Witek et al., 2014; Fitch, 2016), and possibly micro-timing deviations (cf. Madison et al., 2011; Davies et al., 2013; Stupacher et al., 2013; Kilchenmann and Senn, 2015). The present study demonstrates that in addition to acoustic characteristics of music, environmental and personal factors influence movement to music as well. Specifically, familiarity with the performer and musical style (Listener-preference) led to increased movement and entrainment, while the live performance (Concert-status) led to a significant increase in movement vigour. Because synchronous movement can lead to prosociality (Hove and Risen, 2009; Wiltermuth and Heath, 2009; Valdesolo et al., 2010; Valdesolo and DeSteno, 2011; Launay et al., 2013; Cirelli et al., 2014b; Trainor and Cirelli, 2015; Rennung and Goritz, 2016; Woolhouse et al., 2016), and because entrainment to music was fostered more by Listener-preference than Concert-status, it is possible that personal factors are more important than environmental factors for generating synchronous movement and subsequent prosociality.

This study adds to the fledgling literature examining music listening in concert settings (Egermann et al., 2011; Shoda and Adachi, 2012, 2015, 2016; Fancourt and Williamon, 2016; Shoda et al., 2016). It provides unique insight into how live music is experienced in ecologically valid conditions, and how that experience is expressed through body movement. Many questions that remain could be addressed in future research in the LIVELab, such as how individual differences in personality affect live concert experiences, how individuals in a concert setting are affected by the movements of those around them, the effects of different musical characteristics (e.g., tempo, instrumentation, presence of improvisation, genre), whether synchronous movements in a concert setting leads to increased prosociality and bonding, and how performers are affected by audiences.

## Data Availability

The raw data supporting the conclusions of this manuscript will be made available on https://zenodo.org/ (search for ‘LIVELab) by the authors, without undue reservation, to any qualified researcher.

## Ethics Statement

This study was carried out in accordance with the recommendations of the Canadian Tri-Council Policy Statement: Ethical Conduct for Research Involving Humans (T), with written informed consent from all subjects. All subjects gave written informed consent in accordance with the Declaration of Helsinki. The protocol was approved by the McMaster University Research Ethics Board.

## Author Contributions

DS involved in data collection and analyses, and the preparation and review of the manuscript. DB involved in the research design, data collection and analyses, and the preparation and review of the manuscript. SL involved in motion data collection, statistical analyses, and review of the manuscript. JB involved in project and research design, recruitment, organisation, and data collection, and review of the manuscript. MW involved in the conception and organisation of the project including artist-Anthem coordination, research design, and review of the manuscript. SM-R involved in recruitment, data collection and review of the manuscript. LT involved in the conception and organisation of the project, research design, review of the statistical analyses, and preparation and review of the manuscript.

## Conflict of Interest Statement

The authors declare that the research was conducted in the absence of any commercial or financial relationships that could be construed as a potential conflict of interest.

## Footnotes

1 Blown Wide Open was first released in 1997. The containing album achieved double platinum sales status (CRIA) and itself achieved a peak position of #8 on the Canadian rock charts.

2 http://LIVELab.mcmaster.ca

3 https://www.youtube.com/watch?v=0h-Js1KtQa4
