# Supplementary material for: How Live Music Moves Us: Head Movement Differences in Audiences to Live Versus Recorded Music
Source: Front Psychol. 2019 Jan 11;9:2682. doi: 10.3389/fpsyg.2018.02682 (PMC6336707; doi:10.3389/fpsyg.2018.02682)

Supplementary Material

How Live Music Moves Us: Head Movement Differences in Audiences to Live Versus Recorded Music

Swarbrick, D.[1,2], Bosnyak, D.[1,2], Livingstone, S. R.[1,2], Bansal, J.[1,2,3], Marsh-Rollo, S.[1,2], Woolhouse, M. H.[2,3], & Trainor, L. J.[1,2,4*]

[1] Department of Psychology, Neuroscience and Behaviour, McMaster University, Hamilton, ON, Canada L8S 4K1

[2] LIVELab, McMaster Institute for Music and the Mind, McMaster University, Hamilton, ON, Canada L8S 4K1

[3] Digital Music Lab, School of the Arts, McMaster University, Hamilton, ON, Canada, L8S 4K1

[4] Rotman Research Institute, Baycrest Hospital, Toronto, ON, Canada M6A 2E1

*** Correspondence:**
Laurel J. Trainor
ljt@mcmaster.ca

1 Supplementary Table 1. Song Tempos

SONG	BEATS PER MINUTE	
	Live Concert	Album Playback
**1. JUST TO KNOW I CAN**	128	127
**2. HOW LONG**	156	153
**3. FOOL**	116	116
**4. ELOUISE**	136	134
**5. FROZEN POND**	115	115
**6. FEEL**	128	128
**7. SECRETS**	100	97
**8. BLOWN WIDE OPEN**	134	132

2 Supplementary Appendix 1. Participant Survey

ID:__________

Science of *Secrets*: Participant Survey

1. Please answer the following questions, on a scale from 1-7

a. Rate how familiar you are with the musical works by Ian (including Big Wreck & Thornley):

- LOW 1 2 3 4 5 6 7 HIGH

b. Rate how much you enjoy Ian Thornley's music:

LOW 1 2 3 4 5 6 7 HIGH

c. Rate how happy you feel right now:

LOW 1 2 3 4 5 6 7 HIGH

d. Rate how excited you feel right now:

LOW 1 2 3 4 5 6 7 HIGH

2. Are you a musician? YES NO

3. What was your favourite part of this event?

- ____

3 Supplementary Figure 1.

Supplementary Figure 1. LIVELab venue and motion capture. The LIVELab is a 106-seat performance venue with exquisite sound control (the 75 speakers and 28 microphones of the Meyer system digitally control reverb characteristics and play sounds in 3-D) designed to examine the interaction between performers and audience members in the ecologically valid contexts of music, dance, and pedagogy. The motion capture system can record the movements of all audience members and musicians simultaneously. The space is also equipped to measure multi-person EEG and other physiological responses. See LIVELab.mcmaster.ca. Bottom: The four reflective markers on the head of each participant are seen in three-dimensional space as rendered in the Qualysis motion capture system. The average of these four markers was defined as head centre of each participant and was used for the analysis of head speed and degree of entrainment.

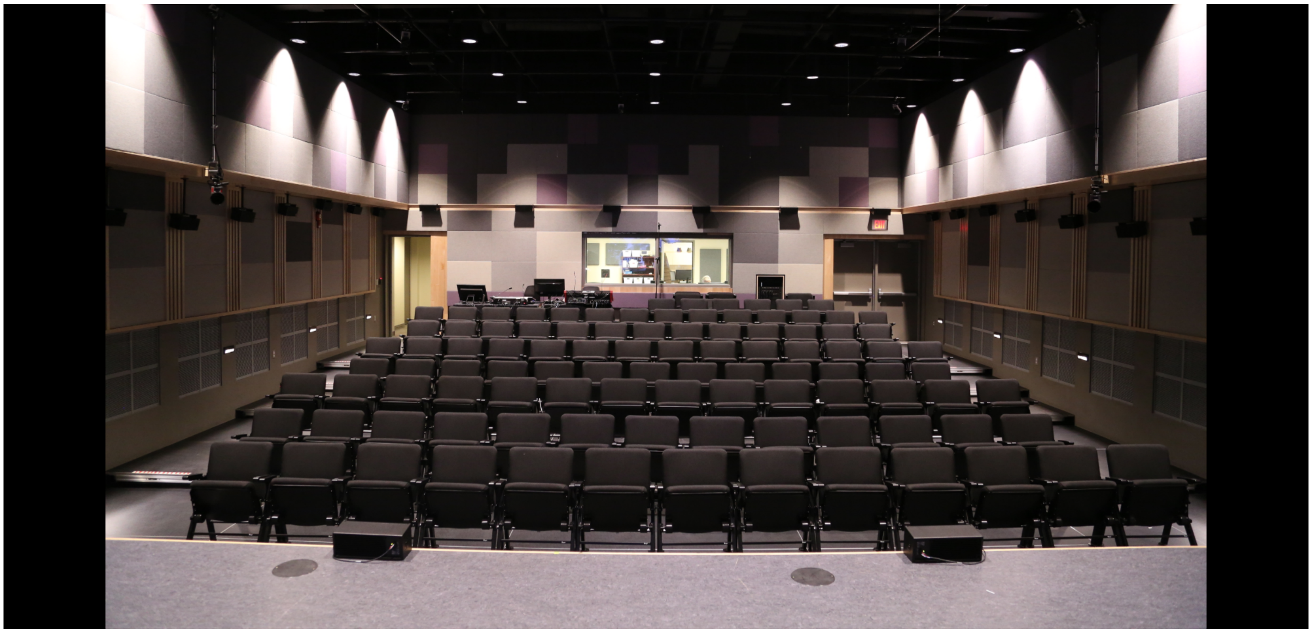

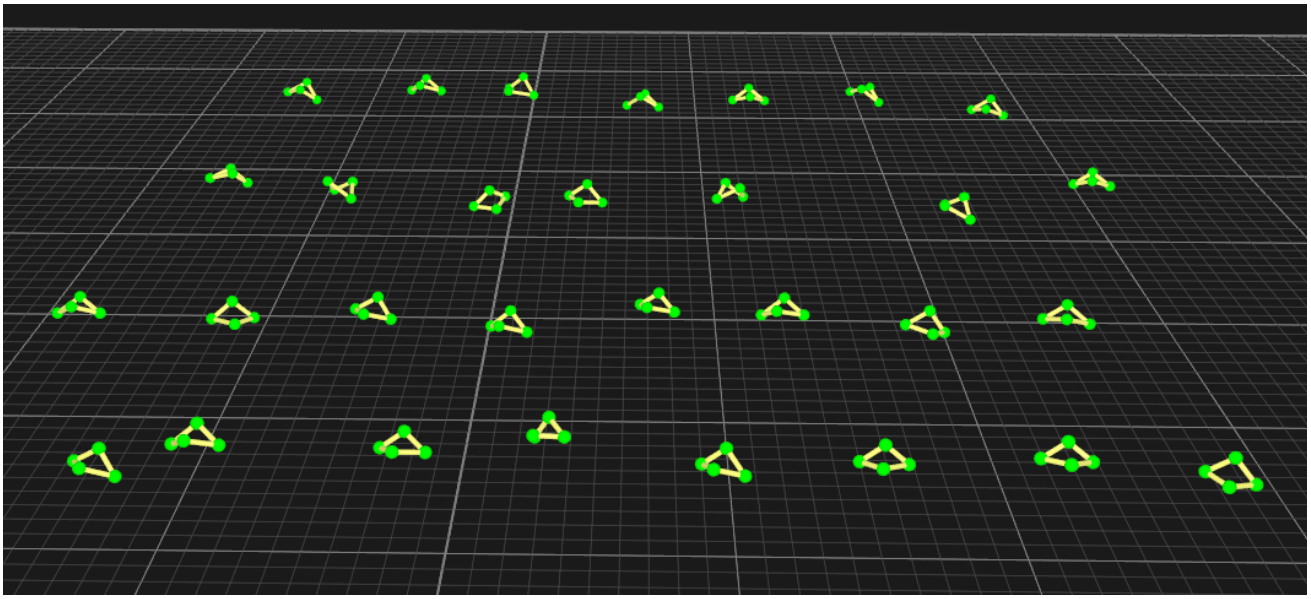

Supplement: Supplementary file 1 [file Data_Sheet_1.PDF]
